# *Synechococcus* sp. PCC7002 Uses Peroxiredoxin to Cope with Reactive Sulfur Species Stress

**DOI:** 10.1128/mbio.01039-22

**Published:** 2022-07-21

**Authors:** Daixi Liu, Jinyu Chen, Yafei Wang, Yue Meng, Yuanning Li, Ranran Huang, Yongzhen Xia, Huaiwei Liu, Nianzhi Jiao, Luying Xun, Jihua Liu

**Affiliations:** a Institute of Marine Science and Technology, Shandong University, Qingdao, People’s Republic of China; b State Key Laboratory of Microbial Technology, Shandong University, Qingdao, People’s Republic of China; c Joint Lab for Ocean Research and Education at Dalhousie University, Shandong University and Xiamen University; d Institute of Marine Microbes and Ecospheres, Xiamen University, Xiamen, China; e School of Molecular Biosciences, Washington State University, Pullman, Washington, USA; Yonsei University

**Keywords:** peroxiredoxin, reactive sulfur species, cyanobacteria, sulfane sulfur reduction

## Abstract

Cyanobacteria are a widely distributed group of microorganisms in the ocean, and they often need to cope with the stress of reactive sulfur species, such as sulfide and sulfane sulfur. Sulfane sulfur refers to the various forms of zero-valent sulfur, including persulfide, polysulfide, and element sulfur (S_8_). Although sulfane sulfur participates in signaling transduction and resistance to reactive oxygen species in cyanobacteria, it is toxic at high concentrations and induces sulfur stress, which has similar effects to oxidative stress. In this study, we report that *Synechococcus* sp. PCC7002 uses peroxiredoxin to cope with the stress of cellular sulfane sulfur. *Synechococcus* sp. PCC7002 contains six peroxiredoxins, and all were induced by S_8_. Peroxiredoxin I (PrxI) reduced S_8_ to H_2_S by forming a disulfide bond between residues Cys^53^ and Cys^153^ of the enzyme. A partial deletion strain of *Synechococcus* sp. PCC7002 with decreased copy numbers of the *prxI* gene was more sensitive to S_8_ than was the wild type. Thus, peroxiredoxin is involved in maintaining the homeostasis of cellular sulfane sulfur in cyanobacteria. Given that peroxiredoxin evolved before the occurrence of O_2_ on Earth, its original function could have been to cope with reactive sulfur species stress, and that function has been preserved.

## INTRODUCTION

Cyanobacteria are one of the most important microbial groups; they provided the first source of O_2_ on Earth via oxygenic photosynthesis (1, 2). However, some environments that cyanobacteria inhabit periodically experience decreased oxygen levels. Cyanobacterial mats are one environment with periodically anoxic conditions, in which cyanobacteria perform oxygenic photosynthesis in the daytime and turn to respiration in the dark. The insufficient diffusion of O_2_ into the mat makes the mat turn anoxic. As a result, heterotrophic bacteria in the mat perform sulfate respiration and produce hydrogen sulfide (H_2_S). Cyanobacteria can use H_2_S as an electron donor to perform anoxygenic photosynthesis when oxygenic photosynthesis is inhibited by high concentrations of H_2_S. Tons of sulfane sulfur may be produced by the oxidation of H_2_S via sulfide:quinone oxidoreductase (SQR) in the mats (3, 4). Sulfane sulfur refers to the various forms of zero-valent sulfur, including persulfide, polysulfide, and elemental sulfur (S_8_). Cyanobacteria inhabiting oxygen minimum zones (OMZs), where H_2_S is sporadically accumulated, also face low-O_2_ and sulfidic conditions. Moreover, cyanobacteria encounter sulfur in the photic zones above OMZs, which are even visible as “clouds” on satellite images. Sulfane sulfur is also likely to be abundant in the benthic realm (5, 6). H_2_S and sulfane sulfur are two of the most important reactive sulfur species (RSS) that tend to be present in sulfidic conditions. RSS are a diverse class of sulfur-containing compounds and functional groups with important roles in chemical biology and bioinorganic chemistry (7–10). Therefore, cyanobacteria need to cope with the RSS stress caused by high concentrations of H_2_S and the accumulation of sulfane sulfur in the environments discussed above (11, 12).

Sulfane sulfur, including persulfide forms (RSSH and HSSH), polysulfide forms (RSS_n_H, RSS_n_R, and H_2_S_n_, *n* ≥ 2), and elemental sulfur (S_8_), is commonly present in the cytoplasm of living organisms and plays important roles in maintaining intracellular redox homeostasis and metabolic regulation (7, 13–15). However, a high concentration of sulfane sulfur is toxic to cells and causes protein persulfidation and disulfide bond formation (16, 17). Inorganic polysulfides generated from organosulfur compounds inhibit several types of pathogenic and drug-resistant bacteria (18). Elemental sulfur is used as a potential antifungal agent (19). Consequently, cells have various enzymes and regulatory systems to protect against excessive levels of sulfane sulfur (20).

In some sulfidic environments, cyanobacteria can perform anoxygenic photosynthesis by using H_2_S as an electron donor, and the key enzyme in this process is SQR (1, 21–23). Cyanobacteria SQR and peroxidase dioxygenase (PDO) can work together to detoxify H_2_S (24). PDO is normally involved in the oxidation of the sulfane sulfur that is produced by SQR, but it functions at high levels of cellular sulfane sulfur. Other pathways that help to maintain the homeostasis of sulfane sulfur in cyanobacteria remain to be explored, considering the important role of sulfane sulfur in cellular signaling (25).

Microorganisms have evolved a series of mechanisms by which to maintain the homeostasis of intracellular sulfane sulfur. Besides PDO (26, 27), thioredoxins (Trx) and glutaredoxins (Grx) also reduce sulfane sulfur to H_2_S (28–31). Furthermore, some cyanobacteria are capable of sulfur respiration, using elemental sulfur as an electron acceptor in dark and anoxic conditions (3, 32, 33). Moreover, catalase, which typically catalyzes the disproportionation of H_2_O_2_ to H_2_O and O_2_ (34, 35), also has the ability to oxidize inorganic persulfide (H_2_S_2_), which is structurally similar to H_2_O_2_ (36). Because peroxiredoxin (Prx) also uses H_2_O_2_ as a substrate (37–39), an immediate question is whether Prx can metabolize sulfane sulfur.

Prxs are ubiquitous in plants, animals, and bacteria (38, 40–43). Their active Cys residue is oxidized to sulfonic acid by H_2_O_2_ and organic peroxides. Depending on whether one or two Cys residues are involved in the process of recycling sulfonic acid back to the thiol form, they can be divided into three categories: typical 2-Cys Prxs, atypical 2-Cys Prxs, and 1-Cys Prxs (40, 44, 45). However, this classification was not unequivocally accepted. Kimberly et al. developed a method that used the Deacon Active Site Profiler tool to extract functional site (PXXXTXXC_P_) profiles from structurally characterized Prxs and classify the Prxs into six distinct subclasses (46, 47): alkyl hydroperoxide reductase subunit C (AhpC-Prx), bacterioferritin comigratory protein (BCP-PrxQ), alkyl hydroperoxide reductase subunit E (AhpE), peroxiredoxin 5 (Prx5), peroxiredoxin 6 (Prx6), and thiol peroxidase (Tpx). Because inorganic polysulfide (H_2_S_n_) and H_2_O_2_ are structural analogs, we hypothesize that Prx can also metabolize H_2_S_n_. From the perspective of evolution, the origin of Prxs precedes the appearance of O_2_ on Earth (48). Therefore, Prx might have been used to manage intracellular RSS before the appearance of O_2_ and may offer cyanobacteria the advantage of being able to move in and out of hypoxic areas in the modern ocean (49–51).

*Synechococcus* sp. PCC7002 (PCC7002) contains six hypothetical Prxs: PrxI (ACA98797.1), PrxII (ACA98565.1), PrxIII (ACA99108.1), PrxIV (ACA98330.1), PrxV (ACA98124.1), and PrxVI (ACA99379.1). Among these, the role of PrxI in H_2_O_2_ metabolism has been confirmed (52). Here, we report that the Prxs in PCC7002 are all induced by S_8_. However, only PrxI was able to reduce S_8_ to H_2_S. The Cys^53^ and Cys^153^ residues of PrxI play critical roles in the reduction of sulfane sulfur to H_2_S via the formation of a disulfide bond. PrxI, which belongs to the Prx5 subfamily, was distinct from the other Prxs of PCC7002 in a phylogenetic analysis. These results improve our understanding of the sulfane sulfur metabolic pathway of cyanobacteria, provide some explanation for the widespread distribution of cyanobacteria in the modern ocean, and provide a new perspective from which to explore the important role of cyanobacteria in the early evolution of life on Earth.

## RESULTS

### Sulfane sulfur upregulates the expression of *prxs* in PCC7002.

**FIG 1:**
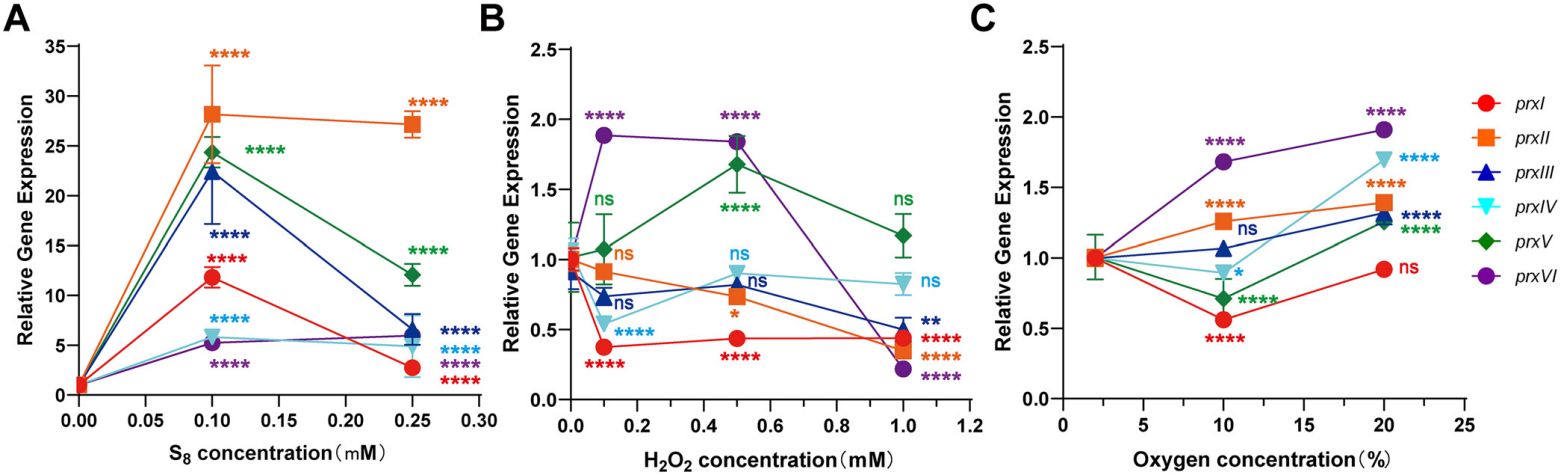
The effects of S_8_, H_2_O_2_, and O_2_ on the expression of *prx*s in PCC7002. The expression levels of *prxI*, *prxII*, *prxIII*, *prxIV*, *prxV*, and *prxVI* were measured using RT-qPCR after induction by S_8_ (A) and H_2_O_2_ (B) for 3 h. (C) The expression of *prxs* in PCC7002 incubated under 2%, 10%, and 20% O_2_ in the gas phase. To determine the expression levels of *prxs*, relative quantitative PCR was used. The relative gene expression represented the *prx* expression levels, standardized by the reference gene *rpnA*. All data are averages from three samples with standard deviations shown (error bars). The experiment was repeated at least three times. *, *P* value < 0.1; **, *P* value < 0.01; ***, *P* value < 0.001; ****, *P* value < 0.0001; ns, not significant (paired *t*-test).

Sulfane sulfur plays an important role in the regulation of the gene expression associated with photosynthesis in PCC7002, but it is toxic at high concentrations (24). S_8_ at the concentrations of 500 μM and 1 mM were fatal to PCC7002 (Fig. S1). Then, 100 and 250 μM S_8_ were used to induce PCC7002, and the expression of *prxs* were detected. All six *prxs* in PCC7002 were upregulated after induction by S_8_, as determined by a quantitative polymerase chain reaction (qPCR) analysis at all tested concentrations (Fig. 1A). At the concentration of 100 μM, the expression levels of *prxIV* and *prxVI* were upregulated by approximately 5-fold. The expression of *prxI* was increased notably (>10-fold). Furthermore, the expressions of *prxII*, *prxIII*, and *prxV* levels were also increased notably (from 20-fold to 30-fold). All *prxs* were also significantly upregulated at the concentration of 200 μM, although the amplitudes were not as high as those observed at 100 μM. Under reactive oxygen species (ROS) pressure, the expression of *prxs* changed slightly, by a maximum of about 2-fold after H_2_O_2_ induction (Fig. 1B) or incubation under 2%, 10%, or 20% O_2_ (Fig. 1C).

10.1128/mbio.01039-22.6 FIG S1 The effect of S_8_ on the growth of PCC7002. (A) The growth curve of PCC7002 in the presence of S_8_. S_8_ at the concentrations of 0, 100, 200, and 500 μM were added to the PCC7002 culture at the beginning of the culturing. (B) The cell morphology of PCC7002 after incubation with 500 μM and 1 mM S_8_. PCC7002 at an OD_730nm_ of 0.05 was cultured for 3 days after the addition of S_8_. All data are averages from three samples with standard deviations shown (error bars). The experiment was repeated at least three times. Download FIG S1, TIF file, 2.0 MB. Copyright © 2022 Liu et al. 2022 Liu et al. https://creativecommons.org/licenses/by/4.0/ This content is distributed under the terms of the Creative Commons Attribution 4.0 International license.

### PrxI metabolized S_8_ and produced H_2_S.

**FIG 2:**
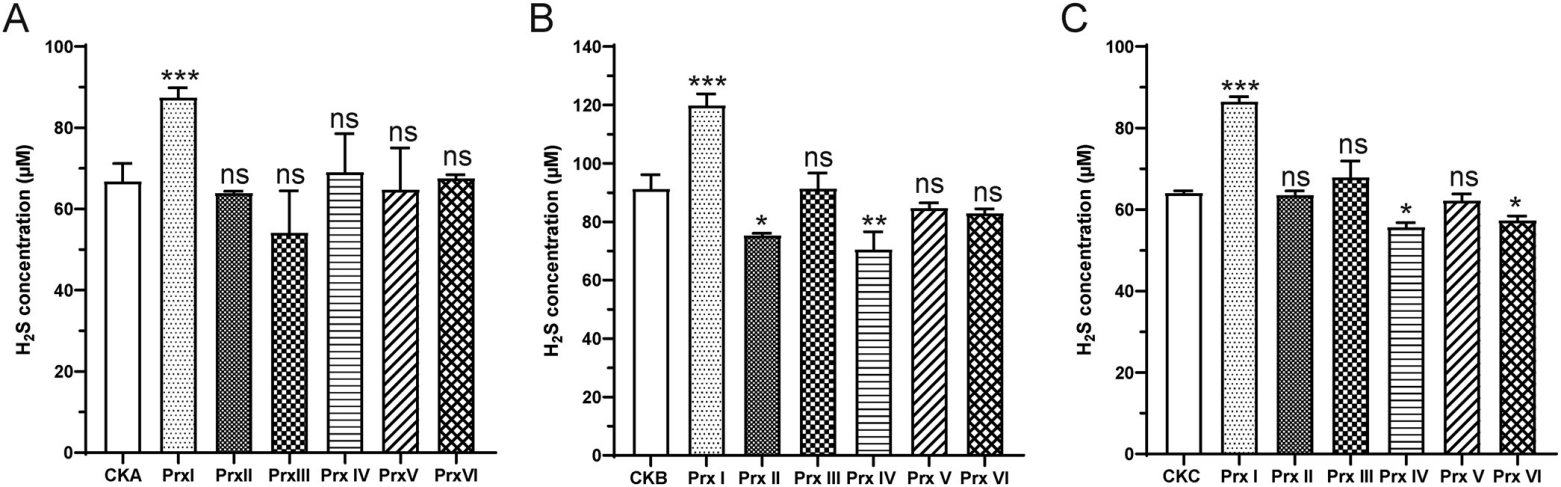
The metabolism of S_8_ by Prxs. (A) The production of H_2_S by purified PrxI-MBP, Prx II-MBP, Prx III-MBP, Prx IV-MBP, Prx V-MBP, and Prx VI-MBP. 100 μg/mL purified Prx-MBP was incubated with 200 μM elemental sulfur (S_8_) and 100 μM DTT for 15 min at 30°C in 50 mM HEPES buffer (pH 7.0). CKA is represented the HEPES buffer with 200 μM elemental sulfur (S_8_) and 100 μM DTT. (B) The production of H_2_S by the lysates of the recombinant *E.coli* BL21 (DE3) (pMal-C2X) (CKB), *E.coli* BL21 (DE3) (pMal-*prxI*) (Prx I), *E.coli* BL21 (DE3) (pMal-*prxII*) (Prx II), *E.coli* BL21 (DE3) (pMal-*prxIII*) (Prx III), *E.coli* BL21 (DE3) (pMal-*prxIV*) (Prx IV), *E.coli* BL21 (DE3) (pMal-*prxV*) (Prx V), and *E.coli* BL21 (DE3) (pMal-*prxVI*) (Prx VI), with a total protein concentration of 10 mg/mL. 200 μM S_8_ was used as the source of sulfane sulfur, and the treatment time was 5 min. (C) The production of H_2_S by recombinant *E.coli* BL21 (DE3) (pMal-C2X) (CKC) and *E.coli* BL21 (DE3) (pMal-*prxI-VI*) cells. The recombinant *E.coli* BL21 strains were harvested and resuspended to an OD_600 nm_ of 10, and then 200 μM S_8_ was added to initiate the reaction. The treatment time was 15 min. All data are averages from three samples with standard deviations shown (error bars). The experiment was repeated at least three times. *, *P* value < 0.1; **, *P* value < 0.01; ***, *P* value < 0.001; ****, *P* value < 0.0001; ns, not significant (paired *t*-test).

To compare the functions of Prxs, H_2_S production by recombinant Prxs fused to the C-terminus of MBP was detected (Fig. 2). Prxs with His-tags were found to be insoluble. First, 100 μg/mL purified Prx–MBP fusion protein (Fig. S2A) were incubated with 200 μM elemental sulfur (S_8_) and 100 μM dithiothreitol (DTT) for 5 min at 30°C in 50 mM HEPES buffer (pH 7.0). About 90 μM H_2_S was released by Prx–MBP, while the H_2_S production by PrxII through PrxVI was not significantly different from that of a control that contained only 200 μM S_8_ and 100 μM DTT in HEPES buffer (Fig. 2A). Second, 200 μM S_8_ were added to cell lysates of recombinant E. coli BL21 expressing Prx–MBP fusion (10 mg of protein mL^−1^). An SDS-PAGE analysis showed a similar amount of the fused proteins in each sample (Fig. S2B). The lysate of recombinant E. coli BL21 expressing the PrxI fusion protein released about 130 μM H_2_S in 5 min of incubation. The control E. coli BL21 with empty vector pMal-C2X released only 90 μM H_2_S. Compared to the control, no more H_2_S was released by the lysates containing PrxII through PrxVI (Fig. 2B). Third, the ability of resting cells expressing Prx–MBP fusion protein to metabolize S_8_ and produce H_2_S was also measured, and only the resting cells with the PrxI-MBP fusion protein produced more H_2_S than did the control cells with the empty vector (Fig. 2C). Thus, PrxI clearly reduced sulfane sulfur to H_2_S.

10.1128/mbio.01039-22.7 FIG S2 SDS-PAGE analysis of the purified Prxs-MBP and the lysates of recombinant E. coli BL21 (DE3) expressing Prxs-MBP. (A) Prxs were fused with MBP on the vector pMal-C2X and expressed in E. coli BL21 (DE3). Prxs-MBP were purified by the amylose resin and resolved by SDS-PAGE. The sample loading was 5 μg of total protein per lane. (B) The cell lysate of the recombinant E. coli BL21 (DE3) was analyzed by SDS-PAGE. The sample loading was 10 μg of total protein per lane. CK, *E.coli* BL21 (pMal-C2X); PrxI, *E.coli* BL21 (pMal-*prxI*); PrxII, *E.coli* BL21 (pMal-*prxII*); PrxIII, *E.coli* BL21 (pMal-*prxIII*); PrxIV, *E.coli* BL21 (pMal-*prxIV*); PrxV, *E.coli* BL21 (pMal-*prxV*); PrxVI, *E.coli* BL21 (pMal-*prxVI*). Download FIG S2, TIF file, 2.9 MB. Copyright © 2022 Liu et al. 2022 Liu et al. https://creativecommons.org/licenses/by/4.0/ This content is distributed under the terms of the Creative Commons Attribution 4.0 International license.

**FIG 3:**
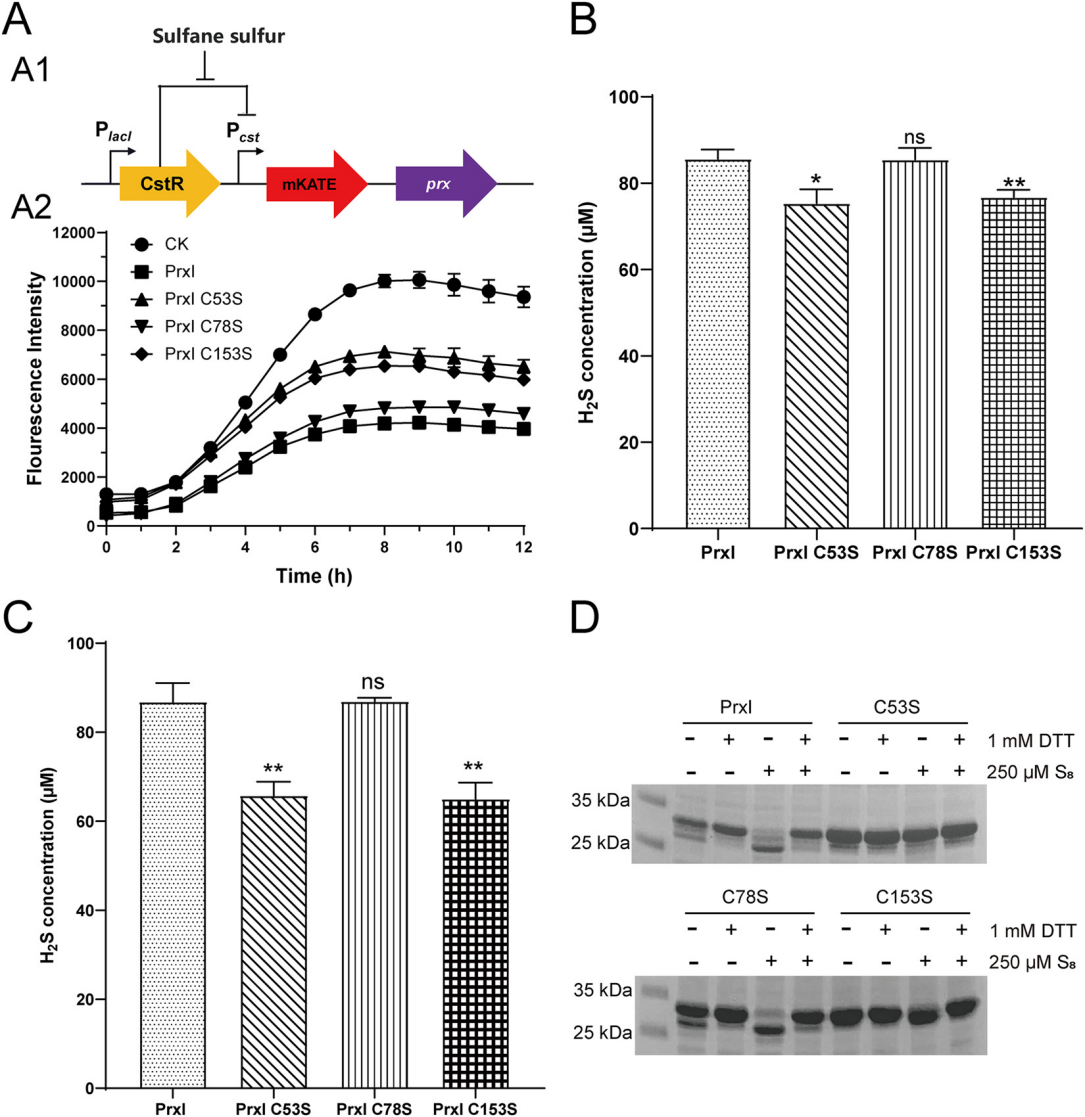
The metabolism of S_8_ by PrxI and its mutants. (A) The function of PrxI detected by the CstR-reporter system. (A1) The schematic diagram of the CstR-reporter system. The CstR reporter system was built to access the activities of Prxs, in which H_2_S_n_ could relieve the repression of *mkate* by CstR. The mKate fluorescence was used to characterize the abilities of Prxs to metabolize sulfane sulfur. (A2) The fluorescence intensities of mKate in the CstR-reporter system coupled with PrxI and its cysteine mutants. (B) The production of H_2_S by purified PrxI, PrxI C53S, PrxI C78S, and PrxI C153S. (C) The production of H_2_S by lysates of recombinant *E.coli* BL21 (DE3) cells expressing *prxI* and its cysteine mutants. (D) Nonreducing SDS-PAGE of PrxI, PrxI C53S, PrxI C78S, and PrxI C153S after S_8_ and DTT treatment. The proteins were cleaved from the MBP-fusion proteins by Factor Xa at room temperature for 24 h, and 6 μg of PrxI proteins were loaded. All data are averages from three samples with standard deviations shown (error bars). The experiment was repeated at least three times. *, *P* value < 0.1; **, *P* value < 0.01; ns, not significant (paired *t*-test).

A previously reported CstR-mKate reporter (53) was adapted to analyze the function of PCC7002 PrxI and its Cys residues. The reporter system included CstR and mKate, in which CstR inhibits the expression of *mkate*. Sulfane sulfur could relieve the inhibitory effect of CstR. Thus, the fluorescence intensity of mKate in the E. coli host cells could serve as an indicator of the levels of intracellular sulfane sulfur (Fig. 3A1), reaching a maximum when E. coli cells entered the early stationary phase (25). When *prxI* was cloned behind *mkate*, the mKate fluorescence was decreased because of the metabolism of sulfane sulfur by PrxI (Fig. 3A2). PrxI contains three cysteine residues (Cys^53^, Cys^78^ and Cys^153^), and they were individually mutated to serine (Ser). The mKate fluorescence intensity in the modified reporter system with PrxI C78S was slightly higher than that in the system with wild-type PrxI. However, the mKate fluorescence intensities with PrxI C53S and PrxI C153S were significantly enhanced compared with those for the construct containing PrxI, and the control without PrxI had the highest mKate fluorescence (Fig. 3A2). Thus, the mutation of Cys^53^ and Cys^153^ destroyed the ability of PrxI to reduce sulfane sulfur.

Furthermore, the Cys residues of PrxI in pMal-C2X were also individually mutated to Ser. Purified PrxI-MBP C53S and PrxI-MBP C153S produced less H_2_S than did wild-type PrxI-MBP and PrxI-MBP C78S, indicating the importance of Cys^53^ and Cys^153^ (Fig. 3B and Fig. S3A). Meanwhile, the lysate of E. coli BL21 cells expressing PrxI-MBP C53S or PrxI-MBP C153S also produced less H_2_S from added S_8_ than did cells expressing wild-type PrxI-MBP or PrxI-MBP C78S (Fig. 3C). The cell lysates of the E. coli BL21 expressing PrxI-MBP and its mutants were standardized by protein concentration and were confirmed to contain similar amounts of proteins by an SDS-PAGE analysis (Fig. S3B).

10.1128/mbio.01039-22.8 FIG S3 SDS-PAGE of the lysates of the purified PrxI, PrxI C53S, PrxI C78S, PrxI C153S, and the recombinant E. coli BL21 (DE3) expressing PrxI and its mutants. PrxI and its mutants were fused with MBP on the vector pMal-C2X and expressed in E. coli BL21 (DE3). (A) PrxsI, PrxI C53S, PrxI C78S, and PrxI C153S were purified by the amylose resin and resolved by SDS-PAGE. The sample loading was 5 μg of total protein per lane. (B) The cell lysate of the recombinant E. coli BL21 (DE3) was analyzed by SDS-PAGE. The sample loading was 10 μg of total protein per lane. PrxI, *E.coli* BL21 (DE3) (pMal-*prxI*); C53S, *E.coli* BL21 (DE3) (pMal-*prxI* C53S); C78S, *E.coli* BL21 (DE3) (pMal-*prxI* C78S); C153S, *E.coli* BL21 (DE3) (pMal-*prxI* C153S). Download FIG S3, TIF file, 2.6 MB. Copyright © 2022 Liu et al. 2022 Liu et al. https://creativecommons.org/licenses/by/4.0/ This content is distributed under the terms of the Creative Commons Attribution 4.0 International license.

We tested whether the Cys^53^ and Cys^153^ of PrxI formed a disulfide bond. The MBP fusion proteins were purified and cleaved by Factor Xa to release PrxI, PrxI C53S, PrxI C78S, and PrxI C153S. The released Prx proteins were analyzed by non-reducing SDS-PAGE. In the SDS-PAGE, untreated PrxI and PrxI C78S showed two bands, with the upper band being dominant. The upper band was converted to the lower band upon treatment with 250 μM S_8_. PrxI C53S and PrxI C153S showed only the upper band, and S_8_ treatment did not affect it (Fig. 3D). The upper band represented the PrxI protein without an intramolecular disulfide bond, while the lower band represented the protein with an intramolecular disulfide bond. All modifications were converted back to thiols by treatment with DTT. Hence, the Cys^53^ and Cys^153^ of PrxI are involved in reducing S_8_ to H_2_S, and they form an intramolecular disulfide bond.

### PrxI enhanced the survival of PCC7002 after sulfane sulfur exposure.

**FIG 4:**
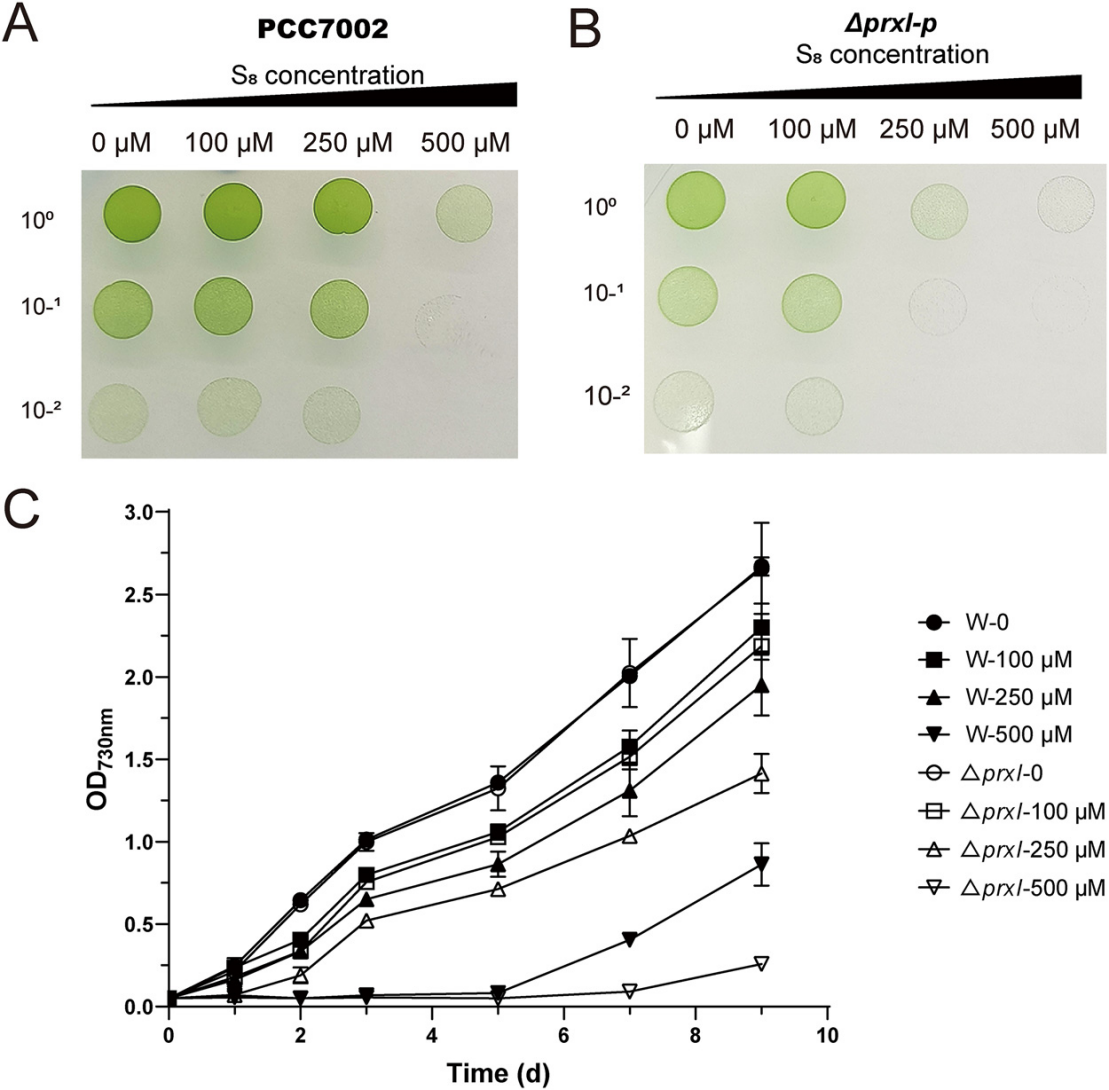
The tolerance of PCC7002 and PCC7002Δ*prxI-*p to S_8_-treatment. The growth of the wild type PCC7002 (A) and its *prxI* partial deletion mutant, PCC7002Δ*prxI*-p, (B) on the A^+^ agar plate after incubation with 100, 250, and 500 μM S_8_. The cells with an OD_730nm_ of 0.05 were treated with S_8_ under 30°C and 50 μmol photons m^−2^ s^−1^illumination for 6 h. Then, the treated cells were diluted with A^+^ medium to 10^0^, 10^−1^, and 10^−2^, then placed on the A^+^ plate for a further cultivation of 7 days under 30°C and 50 μmol photons m^−2^ s^−1^ illumination. (C) The growth curve of PCC7002 and PCC7002Δ*prxI*-p in the presence of S_8_. PCC7002 showed a higher resistance to S_8_ treatment than did PCC7002Δ*prxI*-p. All data are averages from three samples with standard deviations shown (error bars). The experiment was repeated at least three times.

The deletion of *prxI* affected the survival of PCC7002 after sulfane sulfur exposure. We tried to construct a single deletion strain by homologous recombination. However, *prxI* could only be partially knocked out (to give strain PCC7002Δ*prxI*-p), as the kanamycin-resistant mutant contained both the intact *prxI* gene and the kanamycin resistance gene when checked by PCR (Fig. S4). Cyanobacteria often have multiple chromosomes per cell (54), and many critical genes cannot be completely deleted from all chromosomes, as that would be fatal to the cell. Because *prxI* could not be completely deleted, PrxI is likely to play an essential physiological role in PCC7002. Even though not all copies of *prxI* were knocked out, the mutant showed a distinct response to S_8_ exposure compared to that of the wild type. PCC7002 and PCC7002Δ*prxI*-p cells were treated with various amounts of S_8_ for 6 h and then placed on A^+^ agar. After culturing for 7 days, PCC7002 grew well at the S_8_ concentration of 250 μM (Fig. 4A), while the growth of PCC7002Δ*prxI*-p was largely inhibited at that concentration (Fig. 4B). At 500 μM S_8_, PCC7002 could grow in small colonies, while PCC7002Δ*prxI*-p was completely inhibited. Furthermore, the growth curves of PCC7002 and PCC7002Δ*prxI*-p were monitored in the presence of 0, 100, 250, and 500 μM S_8_ (Fig. 4C). The growth curve of PCC7002 and PCC7002Δ*prxI*-p was similar in the absence of S_8_. PCC7002 grew better than PCC7002Δ*prxI*-p did in the presence of 100, 250, and 500 μM S_8_. The above results indicate that PrxI plays a critical role in the survival of PCC7002 after exposure to S_8_.

10.1128/mbio.01039-22.9 FIG S4 The partial deletion of *prxI* was verified by PCR. The *prxI* gene was partially deleted from the PCC7002 genome, displaying two lanes in the agarose gel. Download FIG S4, TIF file, 2.3 MB. Copyright © 2022 Liu et al. 2022 Liu et al. https://creativecommons.org/licenses/by/4.0/ This content is distributed under the terms of the Creative Commons Attribution 4.0 International license.

### Phylogenetic analysis of Prxs in PCC7002.

**FIG 5:**
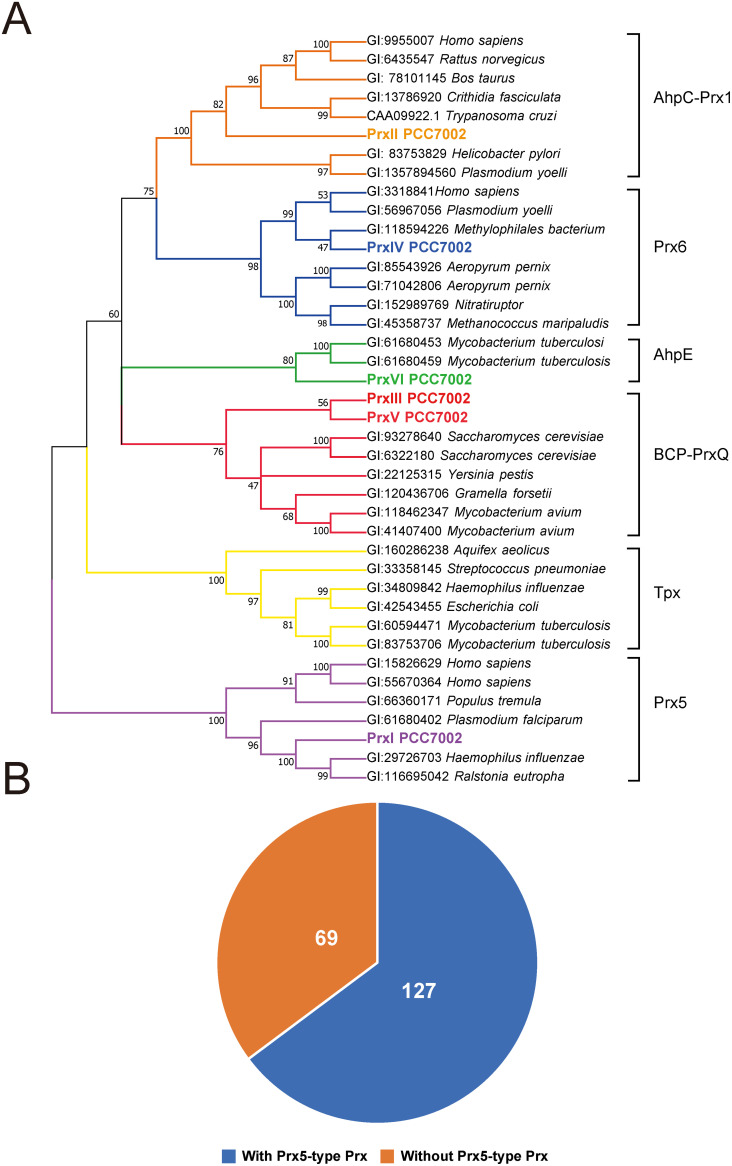
Phylogenetic analysis of Prxs in Cyanobacteria. (A) The genetic diversity of Prxs in PCC7002. The six Prxs in PCC7002 were divided into five subclasses: PrxI PCC7002 belonged to the Prx5 family, PrxII PCC7002 belonged to the AphC-Prx1 family, PrxIII PCC7002 and PrxV PCC7002 belonged to the BCP-PrxQ family, PrxIV PCC7002 belonged to the Prx6 family, and PrxVI PCC7002 belonged to the AphE family. The tree was an unrooted one. The candidates were analyzed by using ClustalW for alignment and MEGA 7.0 for neighbor-joining tree building with the following parameters: pairwise deletion, p-distance distribution, and bootstrap analysis of 1,000 repeats. (B) The fractions of cyanobacteria that carry the Prx5-type Prx. 127 of the 198 cyanobacteria genomes encoded Prx5-type Prx.

We conducted a phylogenetic analysis of the six Prxs in PCC7002 (Fig. 5A). Based on an analysis using the Deacon Active Site Profiler tool, Prxs are classified into six subfamilies (47). Here, representative sequences from each subfamily were selected to analyze the classification of the Prxs in PCC7002 (Table S3). The Prxs in PCC7002 belonged to five subfamilies: PrxI belonged to the Prx5 subfamily, PrxII belonged to the AhpC-Prx1 subfamily, PrxIII and PrxV belonged to the BCP-PrxQ subfamily, PrxVI belonged to the AhpE subfamily, and PrxIV belonged to the Prx6 subfamily. There is no Prx in PCC7002 belonging to the Tpx subfamily. Although the Prx5 subfamily was significantly different from the other subfamilies, as evidenced by it occupying a separate branch in the phylogenetic tree (Fig. 5A), the sequence around the active site of Prx5 subfamily members (PXXXTXXC_P_, where C_P_ is the Cys^53^ of PrxI) is highly conserved (Fig. S5A). Based on the above findings, we deduced that the sequence specificity of PrxI determined its activity.

10.1128/mbio.01039-22.3 TABLE S3 The queries used in the phylogenetic analysis. Download Table S3, DOCX file, 0.02 MB. Copyright © 2022 Liu et al. 2022 Liu et al. https://creativecommons.org/licenses/by/4.0/ This content is distributed under the terms of the Creative Commons Attribution 4.0 International license.

10.1128/mbio.01039-22.10 FIG S5 The consensus sequence and phylogenetic analysis of Prx5-type Prxs in cyanobacteria. (A) Prediction of the conserved sequence of the Prx5-type Prxs active sites by WebLogo. (B) There were 129 Prx5-type Prxs in PCC7002. All of the candidates were analyzed by using ClustalW for alignment and MEGA 7.0 for neighbor-joining tree building with the following parameters: pairwise deletion, p-distance distribution, and bootstrap analysis of 1,000 repeats. Trxs (Thioredoxin) in PCC7002 were used as the outgroup of Prx. Download FIG S5, TIF file, 2.3 MB. Copyright © 2022 Liu et al. 2022 Liu et al. https://creativecommons.org/licenses/by/4.0/ This content is distributed under the terms of the Creative Commons Attribution 4.0 International license.

Currently, 198 genomes of cyanobacteria have been sequenced, and we searched them for Prxs, using the queries in Table S3. There were 1,272 probable Prxs in these cyanobacteria, of which 194 belonged to the AhpC-Prx subfamily, 148 to the Prx6 subfamily, 189 to the AhpE subfamily, 612 to the BCP-PrxQ subfamily, and 129 to the Prx5 subfamily (Table S4). No Tpx family members were found in these cyanobacteria. The 129 Prx5 subfamily members were distributed across 127 cyanobacteria, and 65.5% of the sequenced cyanobacteria encoded at least one Prx5 (Fig. 5B and Fig. S5 and Table S5).

10.1128/mbio.01039-22.4 TABLE S4 The distribution of Prxs in cyanobacteria. Download Table S4, DOCX file, 0.02 MB. Copyright © 2022 Liu et al. 2022 Liu et al. https://creativecommons.org/licenses/by/4.0/ This content is distributed under the terms of the Creative Commons Attribution 4.0 International license.

10.1128/mbio.01039-22.5 TABLE S5 The information of all Prx5-type Prxs in cyanobacteria. Download Table S5, XLSX file, 0.03 MB. Copyright © 2022 Liu et al. 2022 Liu et al. https://creativecommons.org/licenses/by/4.0/ This content is distributed under the terms of the Creative Commons Attribution 4.0 International license.

## DISCUSSION

**FIG 6:**
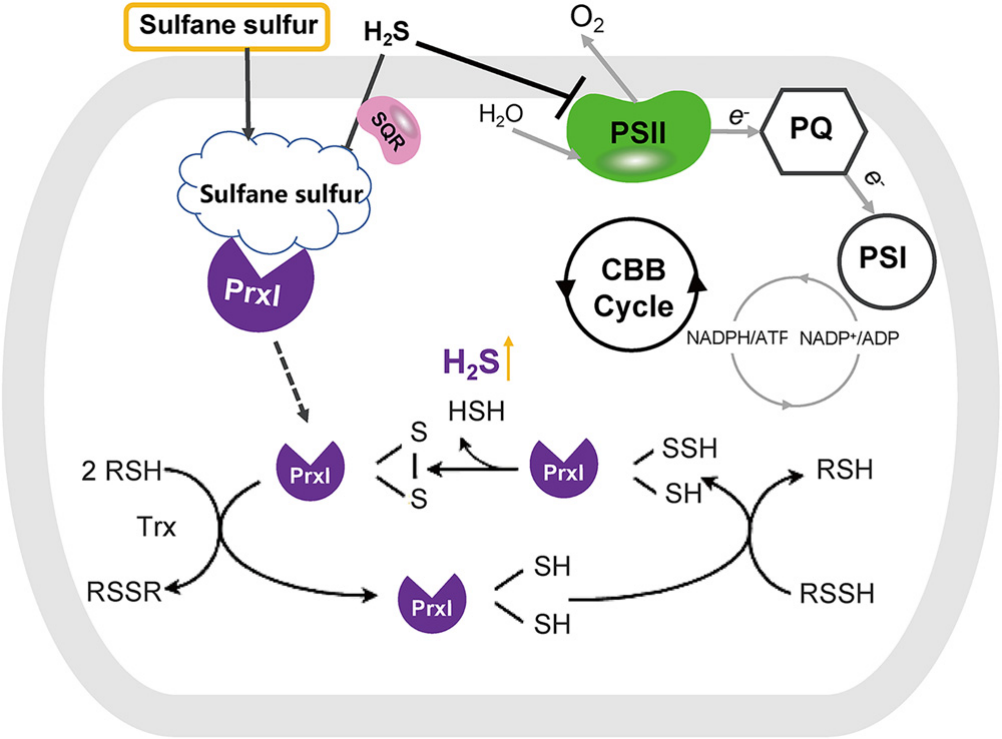
PeroxiredoxinI metabolized S_8_ and produced H_2_S. The expressions of *prxs* in PCC7002 are induced by S_8_. Among these, PrxI reduces S_8_ to H_2_S by donating an electron, thereby generating a disulfide bond between Cys^53^ and Cys^153^. Thioredoxin (Trx) then reduces the disulfide bond. As a result, PrxI helps PCC7002 to cope with the reactive sulfur species stress in the living environments.

Here, we report the participation of PrxI in sulfane sulfur metabolism in cyanobacteria (Fig. 6). S_8_ significantly induced the expression of all six *prxs* in PCC7002 (Fig. 1). Among them, we demonstrated that PrxI reduced S_8_ to H_2_S (Fig. 2) via the formation of an intramolecular disulfide bond between its Cys^53^ and Cys^153^ residues (Fig. 3). When *prxI* was partially inactivated, the PCC7002 mutant became more sensitive to S_8_ (Fig. 4). These results support the idea that PCC7002 uses PrxI to deal with RSS stress.

Three experiments were designed to prove the function of Prxs in PCC7002: one using the purified Prxs-MBP (Fig. 2A), one using the cell lysate of E. coli expressing Prxs-MBP (Fig. 2B), and one using the resting cells of recombinant E. coli expressing Prxs-MBP (Fig. 2C). All three experiments indicated that only PrxI had the ability to reduce S_8_. DTT was used as a reductant in the purified protein experiment. Even though DTT can directly react with S_8_ to produce H_2_S, the existence of PrxI in the reaction produced more H_2_S (Fig. 2A). The maximum production of H_2_S by purified PrxI was at 15 min, while that of the cell lysate was at 5 min. This may be due to the fact that DTT was a chemical reductant which could be much lower than the physiological reductant in the cell lysate (55). The maximum production of H_2_S by the recombinant E. coli with PrxI-MBP was also at 15 min, which may be due to the slow transformation of S_8_ to cells. Furthermore, the lysates of E. coli expressing PrxII-MBP and PrxIV-MBP, as well as the resting cells expressing PrxIV-MBP and PrxVI-MBP, had lower H_2_S production. That may be due to the interaction of Prx with cellular components, as H_2_S production was not decreased in the experiment with using purified proteins (Fig. 2A). Prxs may have the ability to metabolize H_2_S in the presence of cellular components. It has been reported that Cu/Zn superoxide dismutase (SOD) catalyzed H_2_S oxidation to form polysulfide (56). We deduced that Prx may also have that ability, and this needs to be explored in a further study.

Prxs are antioxidant enzymes that play an important role in redox homeostasis and in redox regulation (28, 29). The mechanism of H_2_O_2_ metabolism by Prxs has been well-studied (38, 43). The “peroxidative” cysteine of the catalytic site (C_P_) attacks H_2_O_2_ and is oxidized to sulfenic acid (C_P_–SOH) in the first step of the catalytic cycle. Then, the resolving cysteine (C_R_) attacks the (C_P_–SOH) to release an H_2_O molecule and form a disulfide bond (C_P_–C_R_). Prxs are divided into three classes based on the way the sulfenic acid (C_P_–SOH) is recycled back to a thiol (C_P_–SH): typical 2-Cys Prxs, atypical 2-Cys Prxs, and 1-Cys Prxs. In the typical 2-Cys Prxs, the C_P_-SOH from one subunit is attacked by the C_R_ from the other subunit, resulting in the formation of an inter-subunit disulfide bond. In the atypical 2-Cys Prxs, both the C_P_ and the C_R_ are contained in the same subunit, and the condensation reaction results in the formation of an intramolecular disulfide bond. The 1-Cys Prxs contain only C_P_ and are without C_R_. The C_P_ and C_R_ residues of PCC7002 PrxI are Cys^53^ and Cys^153^, and they formed an intramolecular disulfide bond. A probable mechanism of sulfane sulfur reduction by PCC7002 PrxI is also proposed (Fig. 6) based on that of H_2_O_2_ metabolism, in which Cys^53^ reacts with sulfane sulfur, such as S_8_, to produce a persulfide (Cys^53^–SSH), and Cys^153^ attacks Cys^53^-SSH to form an intramolecular disulfide bond (Cys^53^–Cys^153^) and release H_2_S. In summary, we deduced that PCC7002 PrxI belongs to the atypical 2-Cys Prx family based on its mechanism (37, 40, 43), while it belongs to the Prx5 subfamily based on the analysis by the Deacon Active Site Profiler tool (Fig. 5A).

All six Prxs in PCC7002 were induced by S_8_ (Fig. 1). However, in this study, only PrxI had the S_8_ reduction activity. According to a phylogenetic analysis (Fig. 5), PrxI belongs to a separate branch from the other Prxs in PCC7002, the Tpx subfamily, while the sequences near the C_P_ of the Tpx family are highly conserved (Fig. S5B), suggesting that this region may be the key site for sulfane sulfur reduction, which is also vital for H_2_O_2_ reduction. It remains to be investigated whether other Prxs, especially the Prx5-type Prxs, reduce sulfane sulfur. Our analysis showed that 65% of cyanobacteria encode Prx5-type Prx (Fig. 5B). These results indicate the widespread and important roles of Prx5-type Prx in cyanobacteria.

Prxs are most likely the primary enzymes responsible for maintaining intracellular sulfane sulfur homeostasis in cyanobacteria in anoxic or hypoxic conditions (21, 57). In aerobic conditions, PDO oxidizes sulfane sulfur to sulfite (24). Because the RSS stress is more severe in hypoxic conditions (5, 12), Prxs may play important roles in sulfane sulfur metabolism in anoxic or hypoxic conditions. The action of Prxs against RSS may have been preserved through evolution, as Prxs existed long before oxygen became abundant on Earth (48).

Prxs are known to participate in ROS metabolism (58). Here, we report that they are also involved in RSS metabolism. Cyanobacteria are the oldest surviving microorganisms, and they have experienced the transformation from an anaerobic environment on Earth to an aerobic environment (59, 60). Given the long history of cyanobacterial sulfur exposure, Prxs were most likely first used to resist RSS (5, 60). Interestingly, many of the strategies used against ROS stress, such as catalase, superoxide dismutase, and OxyR, have also been shown to be involved in coping with RSS stress (20, 36, 56). Here, the expression levels of *prxs* in PCC7002 were not as sensitive to H_2_O_2_ induction as they were to induction by S_8_ (Fig. 1C), which might be due to of the activity of other H_2_O_2_ mitigating enzymes, such as catalase and superoxide dismutase. Furthermore, sulfane sulfur might also disturb H_2_O_2_ homeostasis by downregulating catalase, thereby affecting the expression pattern of *prx* (Fig. 1). In summary, there is a close relationship between the strategies for coping with ROS and RSS (15, 61).

The maintenance of sulfane sulfur homeostasis in cyanobacteria is of great importance. In aerobic conditions, sulfane sulfur is an important intracellular signaling molecule that is involved in the regulation of critical photosynthesis genes in cyanobacteria. Sulfane sulfur reduction by Prx would help maintain the normal physiology and photosynthesis of cyanobacteria (24). In hypoxia and darkness, elemental sulfur can be used as an electron receptor for sulfur-dependent respiration, which enables cyanobacteria to yield ATP via the fermentation of endogenous stored glycogen (3, 33). However, high concentrations of sulfane sulfur in this environment can also be toxic to cells, so Prx-mediated sulfane sulfur reduction is a key pathway for detoxification, as photosynthesis ceases and oxidation by PDO is excluded in hypoxia and darkness.

In summary, here, a Prx is shown for the first time to act as a sulfur reductase that reduces S_8_ to H_2_S. Also, cyanobacteria may use Prxs to deal with RSS stress. S_8_ induced the expression of *prxs* in PCC7002, and PrxI worked effectively to reduce S_8_ to H_2_S, thereby improving the tolerance of PCC7002 to S_8_. The conserved sequence of PrxI near residue C_P_ appears to be important for the activity of PrxI. Sulfane sulfur metabolism by Prxs could be the main strategy by which ancient cyanobacteria coped with RSS stress, which facilitated the survival of cyanobacteria in complex environments, especially oxygen-limited areas in the modern oceans.

## MATERIALS AND METHODS

### Strains and culture conditions.

PCC7002 and its mutants were grown in conical flasks containing medium A (62), supplemented with 1 mg of NaNO_3_ mL^−1^ (designed as medium A^+^) under continuous illumination by 50 μmol photons m ^−2^ s ^−1^ at 30°C. Kanamycin (50 μg/mL) was used to select the *prxI* mutant. To explore the effect of O_2_ concentrations, we cultured PCC7002 by bubbling with a mixture of O_2_ and N_2_, with O_2_ contents of 2%, 10%, and 20%. Escherichia coli (E. coli) was cultured in Luria-Bertani (LB) medium at 37°C. The strains and plasmids used in this paper are listed in Table S1.

10.1128/mbio.01039-22.1 TABLE S1 Strains and plasmids used in this study. Download Table S1, DOCX file, 0.02 MB. Copyright © 2022 Liu et al. 2022 Liu et al. https://creativecommons.org/licenses/by/4.0/ This content is distributed under the terms of the Creative Commons Attribution 4.0 International license.

### Induction, RNA extraction, and qRT-PCR analysis.

PCC7002 cells in logarithmic growth with an OD_730 nm_ value of 0.6 to 0.7 were induced by S_8_, H_2_O_2,_ and O_2_ under continuous illumination by 50 μmol photons m^−2^ s^−1^ at 30°C for 3 h. The cells were then harvested by centrifugation at 10,000 × *g* at 4°C for 10 min. Total RNA was isolated using the TaKaRa MiniBEST Universal RNA Extraction Kit, and the concentration of RNA was verified using a Qubit 4 instrument (Thermo Fisher). cDNA was produced using the Prime Script RT Reagent Kit with gDNA Eraser (TaKaRa, Beijing, China). The SYBR Premix *Ex Taq* II Kit (TaKaRa) was used for a quantitative reverse transcriptase polymerase chain reaction (qRT-PCR), and the reactions were run in a Light Cycler 480 II sequence detection system (Roche, Shanghai, China). Primers for target genes are given in Table S2. *rnpA* (SYNPCC7002_A0989), encoding the protein subunit of RNase P (RNase P), was used as the reference gene (63). The results were analyzed according to the 2^−ΔΔCT^ method (64).

10.1128/mbio.01039-22.2 TABLE S2 Primers used in this study. Download Table S2, DOCX file, 0.02 MB. Copyright © 2022 Liu et al. 2022 Liu et al. https://creativecommons.org/licenses/by/4.0/ This content is distributed under the terms of the Creative Commons Attribution 4.0 International license.

### Overexpression of Prxs and enzyme activity determination.

Recombinant Prxs were fused to the C-terminus of maltose binding protein (MBP) and were overexpressed using the vector pMal-C2X (65, 66). Whole fragments encoding *prxI–VI* were amplified from PCC7002 genomic DNA using primers pMal-*prxI–VI*-F/R. Then, the fragments were ligated with pMal-C2X and transformed into E. coli DH5α. The resulting plasmids were transformed into E. coli BL21 (DE3) to overexpress the recombinant Prx fusion proteins. E. coli BL21 (pMal-C2X) and E. coli BL21 (pMal-*prxs*) were cultured in LB at 37°C to an OD_600 nm_ of 0.6. Next, 0.5 mM isopropyl β-D-1-thiogalactopyranoside (IPTG) was added, and the cells were further cultivated at 30°C for 6 h. For resting cell analysis, cells were collected and resuspended in phosphate-buffered saline (PBS; 50 mM, pH 7.4) at an OD_600 nm_ of 10. Then, S_8_ (200 μM) was added to initiate the reaction, and the release of H_2_S was determined by the methylene blue method (67). Here, the S_8_ was made by dissolving sulfur powder in acetone, in which it was soluble in the range of concentrations we used. For the analysis of cell lysates, the collected cells in PBS were disrupted using a pressure cell homogenizer (SPCH-18; Stansted Fluid Power Ltd., United Kingdom). The total protein content in the cell lyses was adjusted to 20 mg mL^−1^, and SDS-PAGE was used to verify whether the lysates contained similar amounts of recombinant Prx. Again, 200 μM S_8_ was added to start the reaction, and the release of H_2_S was determined by the methylene blue method. For the analysis of the purified protein, induced cells were harvested and resuspended in binding buffer (20 mM Tris-HCl, 200 mM NaCl, 1 mM EDTA). Then, the cells were disrupted using a pressure cell homogenizer, and the mixture was centrifuged at 20,000 × *g* for 20 min to acquire crude cell extract. The crude extract was loaded onto amylose resin, and the target protein was eluted using the binding buffer containing 10 mM maltose. The eluted protein solution was then loaded onto a PD-10 desalting column (GE) for buffer exchange to desalting buffer (20 mM NaH_2_PO_4_, 10% glycerol, pH 7.6). The purified proteins were then resolved by SDS-PAGE. The reaction mixtures contained 100 μg/mL Prx, 100 μM DTT, 200 μM S_8_, and 50 mM HEPES-NaOH (pH 7.0). The control containing DTT and S_8_ but no Prx was included. The cysteine to serine mutants of PrxI were generated using the primer pairs *prxI*-C53S-F/R, *prxI*-C78S-F/R, and *prxI*-C153S-F/R with a modified QuikChange site-directed mutagenesis method (68, 69). The reduction of S_8_ by cell lysates of E. coli BL21 (pMal-*prxI* C53S), E. coli BL21 (pMal-*prxI* C78S), and E. coli BL21 (pMal-*prxI* C153S) was detected as described above.

### Non-reducing SDS-PAGE.

PrxI, PrxI C53S, PrxI C78S, and PrxI C153S with the MBP tag were purified in the same way as described above. PrxI, PrxI C53S, PrxI C78S, and PrxI C153S were released from the fusion with MBP by using Factor Xa at room temperature for 24 h. The released proteins were treated with 250 μM S_8_ at 25°C for 30 min. After the S_8_ treatment, 1 mM DTT was added to convert the modified thiols back to reduced thiols. No treatment and treatment with only 1 mM DTT were used as controls. The samples were then resolved by nonreducing SDS-PAGE, in which the loading buffer contained no DTT or other reducing agents.

### Construction of the CstR reporter system.

A CstR-based reporter plasmid was constructed by following a reported protocol to assess the ability of PrxI to metabolize sulfane sulfur (53). In Staphylococcus aureus, CstR (Copper-sensing operon repressor [CsoR]-like sulfurtransferase repressor) is a transcriptional repressor that represses the expression of the *cst* operon, which encodes a putative sulfide oxidation system, by binding to the OP1 and OP2 sites of the *cst* promoter (70). Here, CstR and the *cst* promoter with OP1 and OP2 sites were used to regulate the expression of *mkate* (encoding a red fluorescent protein, mKate). CstR represses the expression of *mkate*, but sulfane sulfur can act on CstR and depress the repression. In this way, the fluorescence intensity of mKate could be used to characterize the concentration of intracellular sulfane sulfur. We constructed plasmids with the *prxI* gene expressed, coupled behind the mKate-encoding gene. The *prxI* gene was cloned using primers cstR-mkate-*prxI*-F/R that contained 20-bp extensions overlapping the vector fragment. Then, the segments were connected with the CstR-OP1-mKate vector by using a TEDA assembly. The three cysteines in PrxI were all individually mutated to serine, using primer pairs *prxI*-C53S-F/R, *prxI*-C78S-F/R, and *prxI*-C153S-F/R, by a QuikChange site-directed mutagenesis to assess their roles. Correct CstR reporter plasmids were transformed into E. coli BL21 for experiments.

### Construction of PCC7002 mutants.

A *prxI* mutant of PCC7002 (PCC7002Δ*prxI-p*) was constructed by homologous recombination as previously reported (24). Briefly, the primer sets *prx*-del-1/*prx*-del-2 and *prx*-del-5/*prx*-del-6 (Table S2) were used to acquire the upstream and downstream segments of the *prxI* gene by PCR from the genomic DNA of PCC7002. The lengths of the segments were about 1,000 bp.

The kanamycin resistance cartridge was amplified from pET30a using primers *prx*-del-3/*prx*-del-4. Long fragments coupling the upstream segment, the kanamycin resistance cartridge, and the downstream segment were obtained by fusion PCR. The fused fragment was connected with the pJET1.2 blunt vector by using the TEDA method (71), and then the resulting vector was transformed into E. coli DH5α by electroporation. Correct transformants were verified by PCR and by sequencing. The correct plasmid was then transformed into PCC7002 by natural transformation. The final transformants were selected using kanamycin (50 μg/mL) and confirmed by PCR.

### Toxicity analysis of sulfane sulfur.

PCC7002 and PCC7002Δ*prxI*-p in the logarithmic growth phase with an OD_730 nm_ of 0.6 to 0.7 were treated with S_8_ for 6 h in sealed centrifugation tubes. After incubation, the cells were washed and resuspended in fresh A^+^ medium. The cells were diluted with A^+^ medium to an OD_730 nm_ of 0.05, and 10 μL were spread on an A^+^-agar plate. Differences between strains PCC7002 and PCC7002Δ*prxI*-p were observed after cultivation at 30°C under continuous illumination by 50 photons m^−2^ s^−1^ for about 7 days. Furthermore, the growth curves of PCC7002 and PCC7002Δ*prxI*-p were also monitored. S_8_ was added to the medium at the beginning of the culturing.

### Phylogenetic analysis.

198 cyanobacterial genomes were downloaded from the NCBI database (updated 17 December 2021). The query sequences of the Prxs were based on reported data (Table S3) (46). The Prx candidates in PCC7002 were analyzed by using ClustalW software for sequence alignment and MEGA 7.0 to build neighbor-joining phylogenetic trees. The parameters were: pairwise deletion, p distance distribution, and bootstrap analysis with 1,000 repeats.

### Data availability.

We will provide any strain and materials used in this study upon request.
