# Supplementary figures and images for: Synechococcus sp. PCC7002 Uses Peroxiredoxin to Cope with Reactive Sulfur Species Stress
Source: mBio. 2022 Jul 21;13(4):e01039-22. doi: 10.1128/mbio.01039-22 (PMC9426444; doi:10.1128/mbio.01039-22)

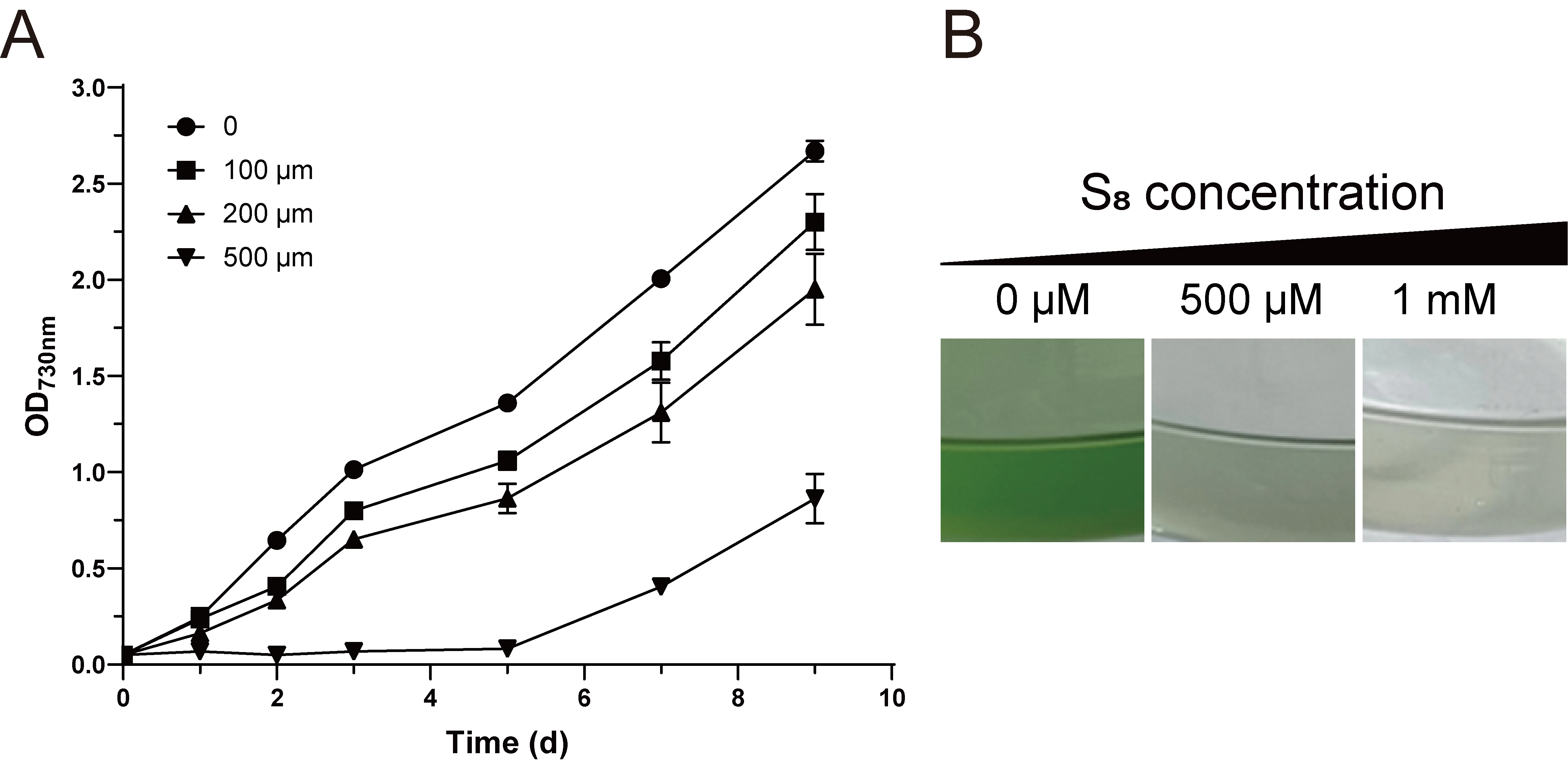

Supplement: FIG S1 [file mbio.01039-22-s0006.tif]

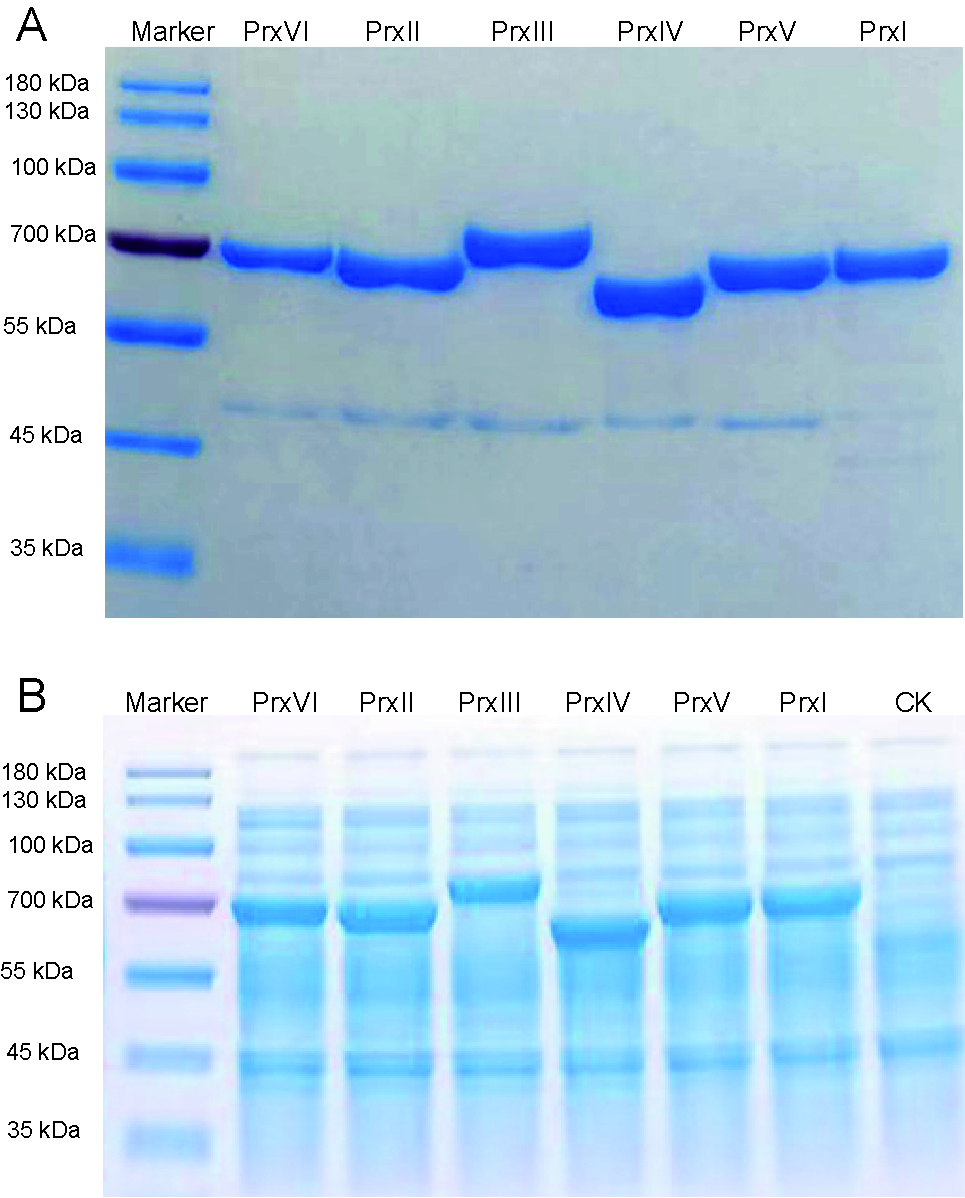

Supplement: FIG S2 [file mbio.01039-22-s0007.tif]

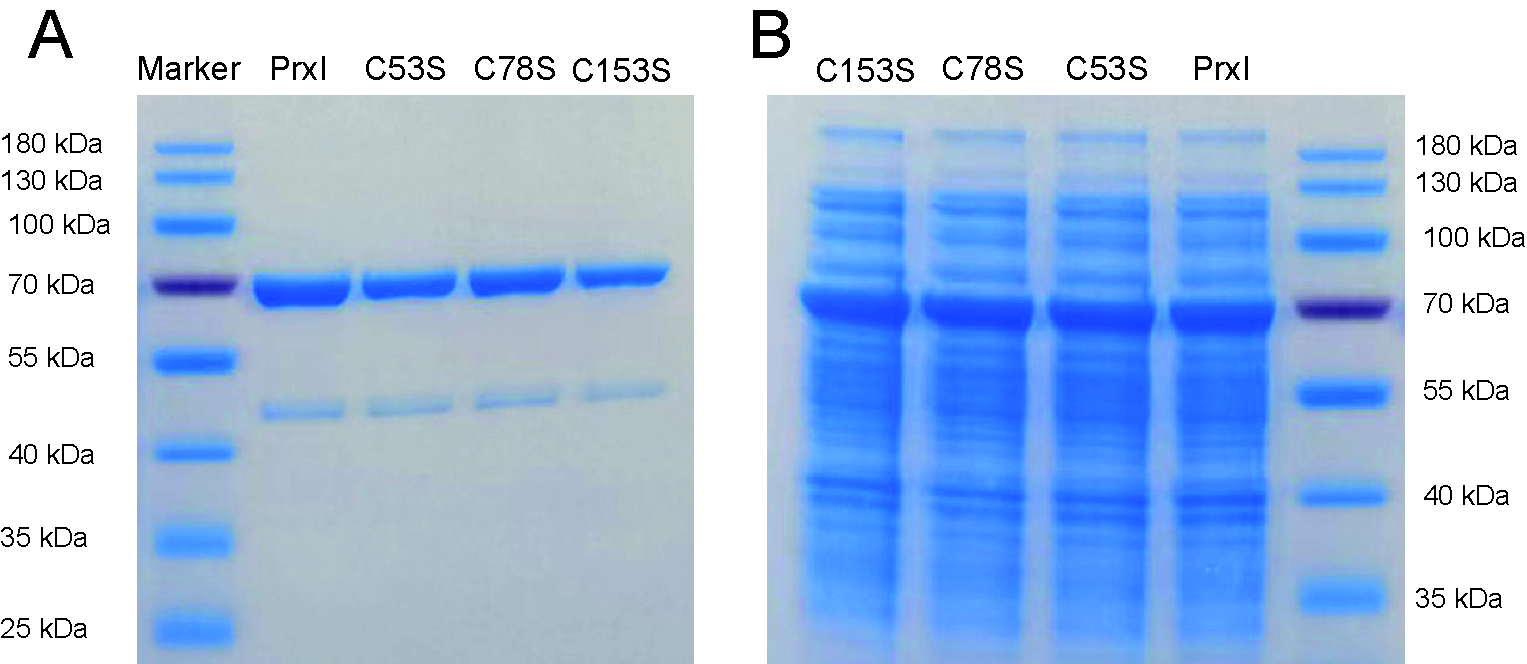

Supplement: FIG S3 [file mbio.01039-22-s0008.tif]

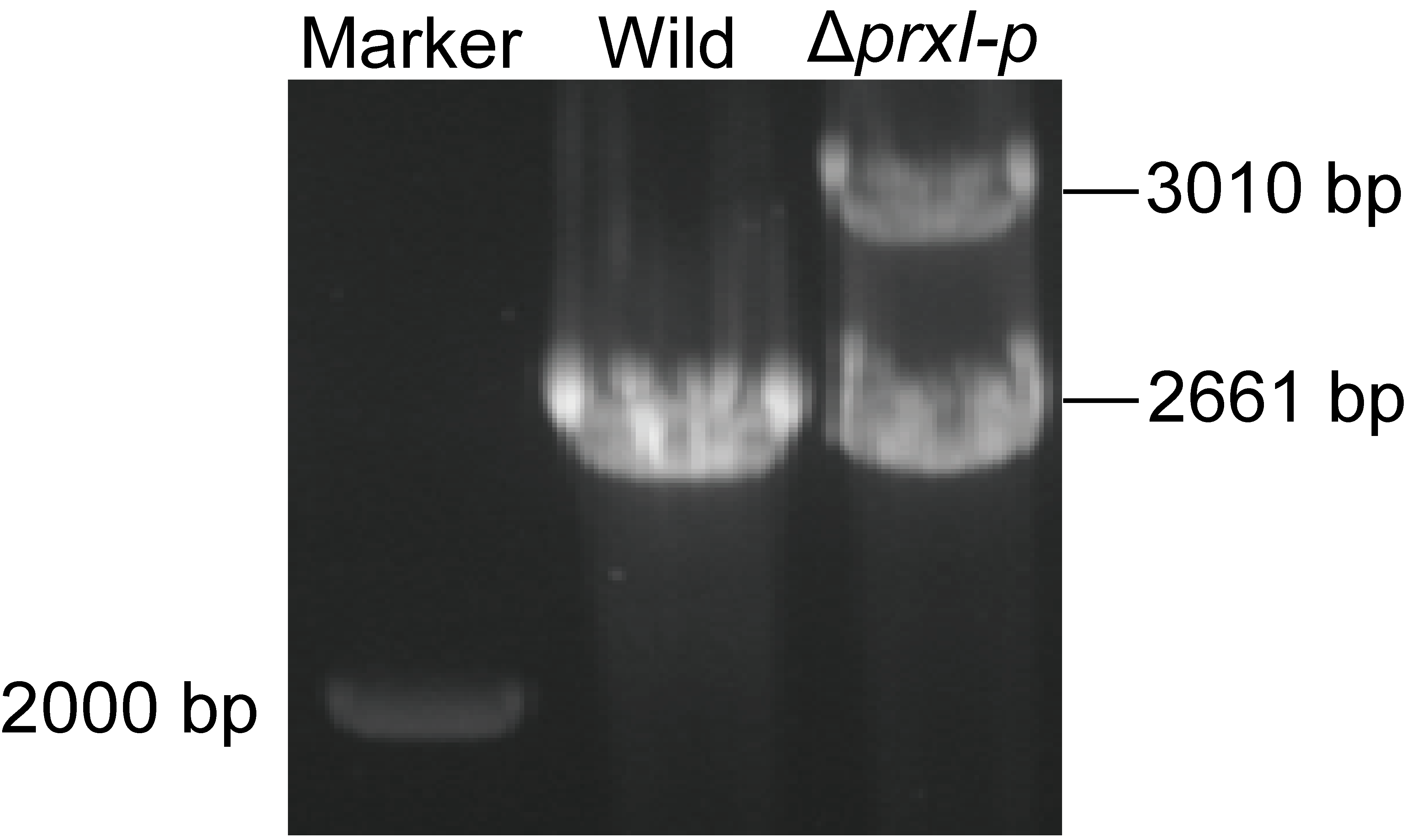

Supplement: FIG S4 [file mbio.01039-22-s0009.tif]

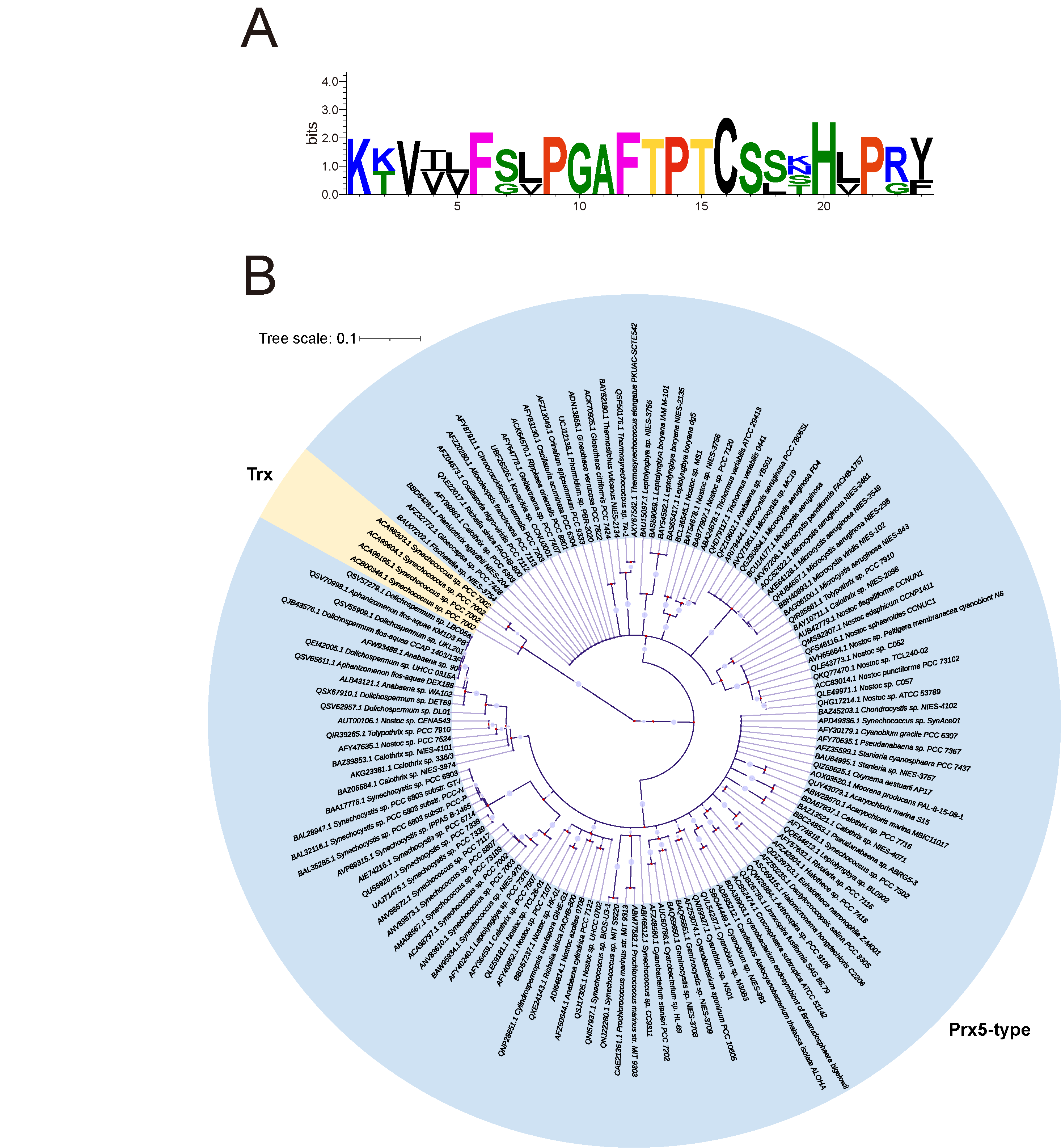

Supplement: FIG S5 [file mbio.01039-22-s0010.tif]
