# Supplementary material for: Synechococcus sp. PCC7002 Uses Peroxiredoxin to Cope with Reactive Sulfur Species Stress
Source: mBio. 2022 Jul 21;13(4):e01039-22. doi: 10.1128/mbio.01039-22 (PMC9426444; doi:10.1128/mbio.01039-22)
Supplement: TABLE S3 [file mbio.01039-22-s0003.docx]

**Table S3 The queries used in the phylogenetic analysis**

| Type | Accession number | Name | Species |
| --- | --- | --- | --- |
| AhpC-Prx1 | 13786920 | tryparedoxin peroxidase | *Crithidia fasciculata* |
|  | 9955007 | Prx2 | *Homo sapiens* |
|  | 6435547 | Prx1 | *Rattus norvegicus* |
|  | CAA09922.1 | tryparedoxin peroxidase | *Trypanosoma cruzi* |
|  | 83753829 | AhpC | *Helicobacter pylori* |
|  | 78101145 | Prx 3 | *Bos taurus* |
|  | 1357894560 | thiol peroxidase 1 | *Plasmodium yoelli* |
| Prx6 | 3318841 | Prx6 | *Homo sapiens* |
|  | 56967056 | 1-Cys Prx | *Plasmodium yoelli* |
|  | 85543926 | thioredoxin peroxidase | *Aeropyrum pernix* |
|  | 71042806 | thioredoxin peroxidase | *Aeropyrum pernix* |
|  | 118594226 | rehydrin | *Methylophilales bacterium* |
|  | 152989769 | alkylhydroperoxide reductase | *Nitratiruptor* |
|  | 45358737 | peroxiredoxin | *Methanococcu s maripaludis* |
| AhpE | 61680453 | AhpE | *Mycobacterium tuberculosis* |
|  | 61680459 | AhpE | *Mycobacterium tuberculosis* |
| BCP-PrxQ | 93278640 | BCP | *Saccharomyces cerevisiae* |
|  | 120436706 | peroxiredoxin | *Gramella forsetii* |
|  | 22125315 | thioredoxin-dependent thiol peroxidase | *Yersinia pestis* |
|  | 118462347 | AhpC/TSA family protein | *Mycobacterium avium* |
|  | 6322180 | Dot5p | *Saccharomyces cerevisiae* |
|  | 41407400 | BcpB | *Mycobacterium avium* |
| Tpx | 33358145 | Thiol peroxidase | *Streptococcus pneumoniae* |
|  | 34809842 | thiol peroxidase | *Haemophilus influenzae* |
|  | 42543455 | thiol peroxidase | *Escherichia coli* |
|  | 60594471 | Tpx | *Mycobacterium tuberculosis* |
|  | 83753706 | Tpx | *Mycobacterium tuberculosis* |
|  | 160286238 | Thiol peroxidase | *Aquifex aeolicus* |
| Prx5 | 15826629 | Prx5 | *Homo sapiens* |
|  | 55670364 | Prx5 | *Homo sapiens* |
|  | 66360171 | PrxD | *Populus tremula* |
|  | 29726703 | PrxV | *Haemophilus influenzae* |
|  | 61680402 | pfAOP | *Plasmodium falciparum* |
|  | 116695042 | Peroxiredoxin | *Ralstonia eutropha* |
