# Supplementary material for: Synechococcus sp. PCC7002 Uses Peroxiredoxin to Cope with Reactive Sulfur Species Stress
Source: mBio. 2022 Jul 21;13(4):e01039-22. doi: 10.1128/mbio.01039-22 (PMC9426444; doi:10.1128/mbio.01039-22)
Supplement: TABLE S4 [file mbio.01039-22-s0004.docx]

**Table S4 The distribution of Prxs in cyanobacteria.**

|  | AhpC-Prx | BCP-PrxQ | AhpE | Prx5 | Prx6 | Tpx |
| --- | --- | --- | --- | --- | --- | --- |
| Number | 194 | 612 | 189 | 129 | 148 | 0 |
| Total | 1272 | | | | | |
