# Supplementary material for: Synechococcus sp. PCC7002 Uses Peroxiredoxin to Cope with Reactive Sulfur Species Stress
Source: mBio. 2022 Jul 21;13(4):e01039-22. doi: 10.1128/mbio.01039-22 (PMC9426444; doi:10.1128/mbio.01039-22)
Supplement: TABLE S1 [file mbio.01039-22-s0001.docx]

**Table S1** **Strains and plasmids used in this study**

| Strain or plasmid | Description/characteristic | Source/reference |
| --- | --- | --- |
| Strains |  |  |
| PCC7002 | Wilde type | This study |
| PCC7002Δ*prxI-p* | PCC7002 with *prx* deletion | This study |
| *E. coli* DH5α | Cloning strain | Novagen |
| *E. coli* BL21(DE3) | Cloning strain | Novagen |
| Plasmids |  |  |
| pMal-C2X | expression vector | 0ur lab |
| pMal-*prxI* | pMal-C2X containing *prxI* | This study |
| pMal-*prxII* | pMal-C2X containing *prxII* | This study |
| pMal-*prxIII* | pMal-C2X containing *prxIII* | This study |
| pMal-*prxIV* | pMal-C2X containing *prxIV* | This study |
| pMal-*prxV* | pMal-C2X containing *prxV* | This study |
| pMal-*prxVI* | pMal-C2X containing *prxVI* | This study |
| pMal-*prxI* C53S | pMal-C2X containing *prxI* C53S | This study |
| pMal-*prxI* C78S | pMal-C2X containing *prxI* C78S | This study |
| pMal-*prxI* C153S | pMal-C2X containing *prxI* C53S | This study |
| pTrchis2A-P*_lacI_*-*cstR*-P*_op1_*-*mkate* | CstR-based reporter for detecting intracellular sulfane sulfur | 0ur lab |
| pTrchis2A-P*_lacI_*-*cstR*-P*_op1_*-*mkate-prxI* | CstR-based reporter with *prxI* | This study |
| pTrchis2A-P*_lacI_*-*cstR*-P*_op1_*-*mkate-prxI* C53S | CstR-based reporter with *prxI* C53S | This study |
| pTrchis2A-P*_lacI_*-*cstR*-P*_op1_*-*mkate-prxI* C78S | CstR-based reporter with *prxI* C78S | This study |
| pTrchis2A-P*_lacI_*-*cstR*-P*_op1_*-*mkate-prxI* C153S | CstR-based reporter with *prxI* C153S | This study |
| pJET1.2-Blunt | Cloning vector | Thermo Fisher |
| pJET-*prxI*-del | *prxI* deletion vector | This study |
