# Supplementary material for: Synechococcus sp. PCC7002 Uses Peroxiredoxin to Cope with Reactive Sulfur Species Stress
Source: mBio. 2022 Jul 21;13(4):e01039-22. doi: 10.1128/mbio.01039-22 (PMC9426444; doi:10.1128/mbio.01039-22)
Supplement: TABLE S2 [file mbio.01039-22-s0002.docx]

**Table S2 Primers used in this study**

| **Primer name** | **Sequence** |  |
| --- | --- | --- |
| *prxI*-*del*-1 | AGCACACATCCCAACAACAGC |  |
| *prxI*-*del*-2 | CCAACTTAATCGCCTTGCAGCAGAGGGTAACAGTGTGGTCGGT |  |
| *prxI*-*del*-3 | ACCGACCACACTGTTACCCTCTGCTGCAAGGCGATTAAGTTGG |  |
| *prxI*-*del*-4 | GTGGGGATTGGTGTGGCTTCATACGACAGGTTTCCCGACTGG |  |
| *prxI*-*del*-5 | CCAGTCGGGAAACCTGTCGTATGAAGCCACACCAATCCCCAC |  |
| *prxI*-*del*-6 | CATTTGTGTAGCGTGAATCAGG |  |
| *prxVI-*qPCR-F | CCAGGATCTAGGCATTGTCG |  |
| *prxVI-*qPCR-R | ATCGCCTGGGACTCATCATA |  |
| *prxII-*qPCR-F | GTAGGCCGTGCTAATGGTTT |  |
| *prxII-*qPCR-R | GTATTGGGCGTTTCCGTAGA |  |
| *prxIII-*qPCR-F | GCTCAACAGTTCGATGGCTA |  |
| *prxIII-*qPCR-R | CCGAAGCCCTAGACTTTACG |  |
| *prxIV-*qPCR-F | ATCGGGTAATTGACAGCACA |  |
| *prxIV-*qPCR-R | GAAGTGGCTCGGTTAAAGGG |  |
| *prxV-*qPCR-F | GCCAATGGGTCGTACTCTAC |  |
| *prxV-*qPCR-R | TGGGAATCGACATCGTCAAC |  |
| *prxI-*qPCR-F | AACGGTGAATTTACCCGCAA |  |
| *prxI-*qPCR-R | CCCTTGATGTAAGCCAGCAT |  |
| *Rnp*A-*-*qPCR -F | GCCCCAACCAAAATTGGCATCAG |  |
| *Rnp*A-*-*qPCR -R | TACCGCACAGCAATGACGATGTG |  |
| *cstR-mkate-prxI-*F | GTTGAGGAAGGAGATTAACTTGGCTAGAGTACCTGACGTTGTCTTC |  |
| *cstR-mkate-prxI-*R | CGTTCACCGACAAACAACAGTTAGCCAACAAATTCTTTGCGGGGTTC |  |
| *prxI-C53S-*F | ACGCCCACTTCTTCCTCCAACCACTTACCCCGTTAC |  |
| *prxI-C53S-*R | GTTGGAGGAAGAAGTGGGCGTGAAAGCACCA |  |
| *prxI-C78S-*F | AATCATCTCTCTGTCTGTAAACGATGCTTTCG |  |
| *prxI-C78S-*R | ACAGACAGAGAGATGATTTCATCAACGCCCTG |  |
| *prxI-C153S-*F | TGATAACTCTCCCACCGATCCTTTTGAAGTATCTGATG |  |
| *prxI-C153S-*R | TCGGTGGGAGAGTTATCAGAGAAGTCAGGCTCTAC |  |
| *pmal-prxVI-*F | CCTCGGGATCGAGGGAAGGATGGCTCGCACTGAATCCAC |  |
| *pmal-prxVI-*R | GTAAAACGACGGCCAGTGCCATTAGCCAAAATAACTGGGGGC |  |
| *pmal-prxII-*F | CCTCGGGATCGAGGGAAGGATGGCCGCACAAGTGGG |  |
| *pmal-prxII-*R | TGTAAAACGACGGCCAGTGCCATTAGAGGGTCGAGAAATAAGCCTG |  |
| *pmal-prxIII-*F | CCTCGGGATCGAGGGAAGGATGACGACCCTGACGATTGG |  |
| *pmal-prxIII-*R | TGTAAAACGACGGCCAGTGCCATTAGGTTTGGAGCGTCTCCAG |  |
| *pmal-prxIV-*F | CCTCGGGATCGAGGGAAGGATGTCTCTCCGGTTAGGCG |  |
| *pmal-prxIV-*R | TGTAAAACGACGGCCAGTGCCATTACTTATTCGGTTGGGGGGTC |  |
| *pmal-prxV-*F | CCTCGGGATCGAGGGAAGGATGTTGCAATTTTTTCGGACGATTC |  |
| *pmal-prxV-*R | TGTAAAACGACGGCCAGTGCCATTAACCCATCGGGGGAAGC |  |
| *pmal-prxI-*F | CCTCGGGATCGAGGGAAGGATGGCTAGAGTACCTGACGTTG |  |
| *pmal-prxI-*R | TGTAAAACGACGGCCAGTGCCATTAGCCAACAAATTCTTTGCGGGGTTC |  |
